# Hyperoside Protects Human Umbilical Vein Endothelial Cells Against Anticardiolipin Antibody-Induced Injury by Activating Autophagy

**DOI:** 10.3389/fphar.2020.00762

**Published:** 2020-05-21

**Authors:** Aiwu Wei, Huidongzi Xiao, Guangli Xu, Xile Yu, Jingjing Guo, Zhuqing Jing, Shaoqi Shi, Yanli Song

**Affiliations:** ^1^Department of Reproductive Medicine, The First Affiliated Hospital of Henan University of Chinese Medicine, Zhengzhou, China; ^2^Department of Reproductive Medicine, Henan Province Hospital of Traditional Chinese Medicine, Zhengzhou, China

**Keywords:** hyperoside, human umbilical vein endothelial cells, anticardiolipin antibody, injury, autophagy

## Abstract

Anticardiolipin antibody (aCL), an important characterization of antiphospholipid syndrome, shows an intense association with vascular endothelial injury. Hyperoside is a flavonoid extracted from medicinal plants traditionally used in Chinese medicines, displaying anti-inflammatory, anti-cancer, and anti-oxidative properties in various diseases. Recent studies have shifted the focus on the protective effects of hyperoside on vascular endothelial injury. However, little is known about the mechanisms involved. In the present study, we investigated the effect of hyperoside on aCL-induced injury of human umbilical vein endothelial cells (HUVECs) *in vitro*. Our data illustrated that aCL induced HUVEC injury *via* inhibiting autophagy. Hyperoside reduced aCL-induced secretion of proinflammatory cytokines IL-1β and IL-8 and endothelial adhesion cytokines TF, ICAM1, and VCAM1 in HUVECs. Additionally, hyperoside activated autophagy and suppressed the mTOR/S6K and TLR4/Myd88/NF-κB signaling transduction pathways in aCL-induced HUVECs. To the best of our knowledge, this is the first study to investigate the effect of hyperoside on aCL-induced injury, as well as offer insights into the involved mechanisms, which is of great significance for the treatment of antiphospholipid syndrome.

## Introduction

Antiphospholipid syndrome (APS) is a rare systemic autoimmune disorder clinically characterized by recurrent thrombosis or pregnancy morbidity in combination with the persistent presence of circulating antiphospholipid antibody (aPL), including anticardiolipin antibody (aCL), anti-β2-glycoprotein I (anti-β2GPI), and lupus anticoagulant (LA) (Woo et al., 2010; Gomez-Puerta and Cervera, 2014; Linnemann, 2018). Various mechanisms have been speculated to contribute to the disease progression regarding to inflammation (Becarevic, 2016), adhesion receptors (Lopez-Pedrera et al., 2017), oxidative stress (Benhamou et al., 2015), and neutrophil extracellular traps (Meng et al., 2017) in APS patients. Usually, APS patients have a greater predisposition to cardiovascular disorders, involving coronary artery disease, myocardial infarction, and stroke (Kolitz et al., 2019). Previous studies have confirmed that aPL isolated from APS patients accelerate thrombosis progression in animal models (Manukyan et al., 2016; Papalardo et al., 2017; Sacharidou et al., 2018), but the involved mechanisms are not yet clearly understood.

Vascular endothelial cells play a pivotal role in maintaining normal physiological functions of the cardiovascular system, which secrete a series of vasoactive substances through autocrine, endocrine, or paracrine pathways to regulate blood flow, vascular wall tension, angiogenesis, and inflammation (Li and Shah, 2004; Feigerlova and Battaglia-Hsu, 2017; Montezano et al., 2017). Due to their barrier functions, vascular endothelial cells are more vulnerable to injury induced by physical or chemical risk factors (Awad et al., 2013). Vascular endothelial cell injury occurs in many clinical events, including angiogenesis, atherosclerosis, thrombosis, hypertension, and heart failure (Cheng et al., 2016; Yang et al., 2018; Yang et al., 2019; Yao et al., 2019; Yi and Gao, 2019). Confirmed evidence has validated that vascular endothelial cell injury induced by aPL plays a cardinal role in APS pathogenesis (Corban et al., 2017). Notably, aCL, an important composition of aPL, is closely associated with numerous thromboembolic phenomena (Cappell, 1994), including esophageal necrosis and perforation. The accumulation of aCL may be a vital threat for endothelial cell injury (Westerman et al., 1992). However, until now, the involving mechanisms are still elusive. Therefore, exploration of underlying mechanisms involved in endothelial injury induced aCL, are urgently needed for the treatment of APS.

Autophagy is an evolutionarily conserved process for eliminating nonessential or dysfunctional organelles in living cells (Rajawat and Bossis, 2008; Dokladny et al., 2015). It is well known that autophagic dysfunction companied by the changes in light chain 3 I/II (LC3 I/II), Beclin 1, and p62 is associated with the pathogenesis of many diseases (Li et al., 2018; Uddin et al., 2019). LC3-II, a lipidated form of LC3, has been indicated as an autophagosomal marker in mammals, and has been applied to study autophagy in multiple inflammatory conditions (Tanida et al., 2004). Beclin 1, a key component of the autophagosome nucleation complex, promotes LC3 conversion and the formation of LC3 puncta (Wu et al., 2018). p62, a classical receptor of autophagy, is a multifunctional protein involved in the autophagosomal degradation (Liu W. J. et al., 2016). When autophagy is disrupted, it usually happens that the expressions of LC3 II and Beclin 1 are decreased but p62 is increased. Some lines of evidence have demonstrated that the autophagic dysfunction in vascular endothelial cells causes endothelial dysfunction and vascular homeostasis disruption, further resulting in the pathogenesis of cardiovascular diseases (Nixon, 2013; Guo et al., 2016; Xu et al., 2018). Additionally, mammalian target of rapamycin (mTOR), a master regulator of cellular metabolism, is a crucial molecule in regulating autophagy (Kim and Guan, 2015). However, there have been no available studies on the effect of autophagy or mTOR on aCL-induced injury, as far as we know.

Hyperoside is a flavonoid glycoside compound mainly found in medicinal herbs, which is a promising agent for disease prevention (Liu Y. H. et al., 2016; Xie et al., 2016; Chen et al., 2018). Previous studies have shown that hyperoside displays anti-oxidative, anti-cancer, and anti-inflammatory properties in many molecular events (Yang et al., 2016; Kong et al., 2020). Importantly, hyperoside has been demonstrated to protect human umbilical vein endothelial cells (HUVECs) against hydrogen peroxide-induced injury (Li et al., 2012). Besides, hyperoside has been verified to mediate autophagy in some cancer cell lines (Fu et al., 2016; Zhu et al., 2017). Thus, it is possible that hyperoside prevents endothelial cells against injury by mediating autophagy.

In this study, we sought to investigate whether hyperoside could protect HUVECs from aCL-induced injury and identify the possible mechanism involved. Our data demonstrated the involvement of hyperoside-induced autophagy in aCL-induced injury of HUVECs and suggested that hyperoside might act as a potential pharmacological strategy for aCL-induced endothelial injury in APS patients.

## Materials and Methods

### Clinical Specimens

All studies were approved by the ethics committee of First Affiliated Hospital of Henan University of Chinese Medicine. Sixteen patients diagnosed as aCL-positive (aCL > 140 GPL) fulfilled the informed consents before sample collection in accordance with the declaration of Helsinki. Blood was collected by phlebotomist venipuncture, and serum was collected by standard methods and stored at –80°C until ready for use. All the sera was pooled together before aCL-IgG extraction was carried out. aCL-IgG fractions were extracted following the following steps: Serum was slowly added with saturated ammonium sulfate to a final mass fraction of 33%, and placed at 4°C overnight for protein precipitation. The supernatant was collected, added with saturated ammonium sulfate to a final mass fraction of 50%, and placed at 4°C overnight. Then the supernatant was discarded and the precipitate was retained, dissolved in PBS, and dialyzed for 2 days. The removal of ammonium sulfate was confirmed by addition of 1% BaCl_2_. The proteins were then concentrated with polyethylene glycol solution for further experiments.

### HUVEC Culture and Treatment

Human umbilical vein endothelial cells (Zhong Qiao Xin Zhou Biotechnology, Shanghai, China) were used throughout this study and maintained in endothelial cell culture medium (Zhong Qiao Xin Zhou) supplemented with 10% fetal bovine serum (Sigma-Aldrich, St Louis, MO, USA) in a humidified atmosphere with the presence of 5% CO_2_. Hyperoside (HPLC grade) was purchased from Chengdu Purechem-Standard Co., Ltd., China. Rapamycin and 3-MA were purchased from MedChemExpress, NJ, USA (Purity of all ≥ 98%). Rapamycin is a specific inhibitor of mTOR (Li et al., 2014) and 3-MA is a widely used autophagy inhibitor (Wu et al., 2013).

For the HUVEC injury assay, HUVECs were treated with aCL-IgG in an adjusted dose and duration as previously described (Simantov et al., 1995). In this study, HUVECs were treated with aCL (200 μg/ml) for 30 min, 1 h, 2 h, and 4 h. To evaluate the effect of hyperoside on aCL-induced injury of HUVECs, cells were pre-treated with hyperoside (10, 20, 50 μM) for 24 h and then treated with aCL (200 μg/ml) for 4 h. 3-MA (10 mM) was added at the same time as hyperoside, while rapamycin (100 nM) was added at the same time as aCL.

### Cell Transfection

HUVECs in the logarithmic growth phase were transfected with 2.5 µg LC3B-red fluorescent protein (RFP)-green fluorescent protein (GFP) plasmids using lipofectamine 2000 (Invitrogen, California, USA) according to the manufacturer's instructions. At 48 h after transfection, the cells were photographed with a fluorescence microscope (BX53, Olympus, Tokyo, Japan).

### Immunofluorescence

Cells were seeded on glass slides, fixed with 4% paraformaldehyde (Sinopharm Chemical Reagent, Beijing, China) for 15 min, and permeabilized with 0.1% TritonX‐100 (Beyotime, Shanghai, China) for 30 min. Then cells were blocked with goat serum (Solarbio, Beijing, China) for 15 min, followed by incubation with anti‐NF‐κB p65 (Proteintech Group, IL, USA) at 1:200 dilution in PBS at 4°C overnight. After three times washed with PBS, cells were incubated with Cy3-labeled fluorescent secondary antibody (Beyotime) at 1:200 dilution in PBS and DAPI (Aladdin Regents, Shanghai, China) was used to stain the nucleus. A fluorescence microscope (BX53, Olympus) was then applied to capture images at 400× magnifications.

### Western Blot

For western blot analysis, cells were lysed in RIPA buffer (Beyotime) containing 1 mM PMSF (Beyotime). The supernatants were collected and protein concentration was detected by the BCA protein assay kit (Beyotime). Proteins were loaded and separated on an 8–15% gradient SDS-PAGE gel and transferred to PVDF membranes (Millipore, MA, USA). After blocking nonspecific binding sites with non-fat milk for 1 h, the membranes were incubated with antibodies against mTOR (Proteintech, 1:500), phospho-mTOR^Ser2448^ (p-mTOR^Ser2448^, ABclonal Biotechnology, Wuhan, China, 1:1,000), p70 S6 Kinase (S6K, ABclonal, 1:3,000), phospho-S6K^Thr389^ (p-S6K^Thr389^, ABclonal, 1:1,000), p62 (ABclonal, 1:2,000), Beclin 1 (Proteintech, 1:400), tissue factor (TF, ABclonal, 1:1,000), intercellular cell adhesion molecule-1 (ICAM1, ABclonal, 1:1,000), vascular cell adhesion molecule-1 (VCAM1, ABclonal, 1:2,000), toll like receptor-4 (TLR4, Proteintech, 1:1,000), myeloid differential protein-88 (MyD88, Proteintech, 1:2,000), nuclear factor kappa-B p65 (NF-κB-p65, 1:1,000), phospho-p65^Ser276^ (p-p65^Ser276^, ABclonal, 1:1,000), phospho-p65^Ser536^ (p-p65^Ser536^, ABclonal, 1:1,000), or β-actin (Santa Cruz, CA, USA, 1:1000). Membranes were then incubated with horseradish peroxidase-labeled secondary antibody at 1:5,000 dilution for 1 h at room temperature. The bands were detected with an imaging system (WD-9413B, Liuyi Biotechnology, Beijing, China). ImageJ software (version 1.51a) was used to analyze band density. The experiment was performed in triplicate.

### Quantitative Real-Time Polymerase Chain Reaction (RT-PCR)

Total RNA was extracted from HUVECs using TRIpure (BioTeke Corporation, Beijing, China) reagent. cDNA was synthesized with Super M-MLV Reverse transcriptase (BioTeke). Real-time PCR was performed using Power SYBR Green PCR Master Mix (BioTeke) on ExicyclerTM^96^ fluorometer (Bioneer Corporation, Daejeon, Korea). mRNA levels were calculated using the 2^−ΔΔCT^ method and normalized to the value of β-actin. The primer sequences were presented in **Table 1**. The experiment was performed in triplicate.

**Table 1:** Primer sequences for qRT-PCR.

Primer	Sequence
IL-1β	F: GAATCTCCGACCACCACTAC
	R: CACATAAGCCTCGTTATCCC
IL-8	F: CACAAACTTTCAGAGACAGCAG
	R: GTGGAAAGGTTTGGAGTATGTC
TF	F: TGTCTACATAGCGGGCAAGT
	R: GTTCCAGCCAGCGGTTCT
ICAM1	F: GCAAGAAGATAGCCAACCAAT
	R: TGCCAGTTCCACCCGTTC
VCAM1	F: GAAATGACCTTCATCCCTAC
	R: GCTGACCAAGACGGTTGTAT
β-actin	F: CTTAGTTGCGTTACACCCTTTCTTG
	R: CTGTCACCTTCACCGTTCCAGTTT

IL-1β, interleukin-1β; IL-8, interleukin-8; TF, tissue factor; ICAM1, intercellular cell adhesion molecule-1; VCAM1, vascular cell adhesion molecule-1.

### Enzyme-Linked Immunosorbent Assay (ELISA)

Concentrations of E-selectin, interleukin-1β (IL-1β) and interleukin-8 (IL-8) in the culture medium were determined using corresponding ELISA kits (USCN Life Science, Wuhan, China) according to the manufactures' protocols. Briefly, samples or standards were added to triplicate microplate wells precoated with corresponding monoclonal antibodies, and incubated for 1 h. The plates were washed three times and incubated with the enzyme-linked polyclonal antibodies for 2 h. Then the wells were washed five times to remove the unbound antibodies, and added with substrate solution. After incubation for 20 min, the enzyme reaction was stopped with stop solution. Optical density values were determined by a microplate reader (ELX-800, BioTek Instruments, VT, USA) and concentrations were calculated according to the standard curve. The experiment was performed in triplicate.

### Data Analysis

Statistical analysis was conducted using GraphPad Prism 8.0.2 Software (Version X, CA, USA). Data were analyzed by one-way analysis of variance (ANOVA) followed by Turkey's *post hoc* tests and presented as mean ± SD. Values of P < 0.05 were considered as statistically significant.

## Results

### Autophagy Was Inhibited in aCL-Induced Injury of HUVECs

**Figure 1:**
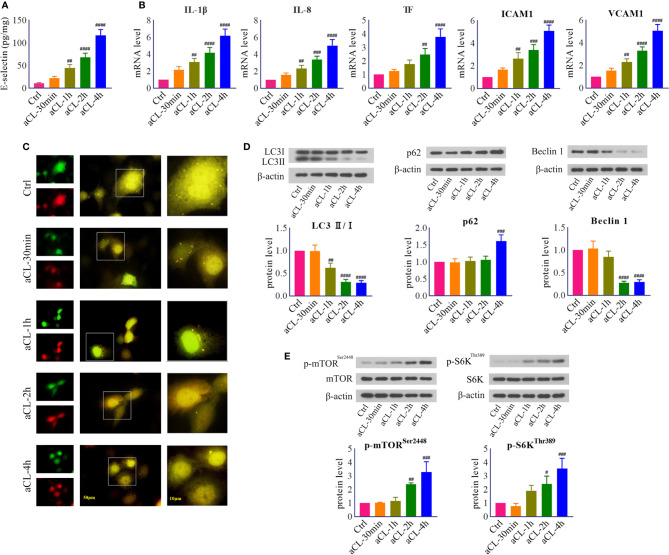
HUVECs were injured by aCL treatment and autophagy was inhibited. HUVECs were treated with aCL (200 μg/ml) for 0, 30 min, 1 h, 2h, and 4 h, respectively. **(A)** ELISA analysis of E-selectin. **(B)** Real-time PCR analysis of IL-1β, IL-8, TF, ICAM1, and VCAM1. **(C)** Fluorescence results in cells transfected with LC3B-RFP-GFP plasmid. **(D)** Western blot analysis of LC3, p62, and Beclin 1 and corresponding gray values of protein bands. **(E)** Western blot analysis of mTOR, p-mTOR^Ser2448^, S6K, and p-S6K^Thr389^ and corresponding gray values of protein bands. Data were presented as mean ± SD, n = 3. ^#^P < 0.05, ^##^P < 0.01, ^###^P < 0.001, ^####^P < 0.0001 *vs.* Ctrl. Ctrl represented Control.

First of all, to evaluate the injury effect of aCL on HUVECs, cells were treated with 200 μg/ml aCL for 0, 30 min, 1 h, 2 h, and 4 h, respectively. The results of ELISA showed that the level of E-selectin was significantly elevated after aCL treatment for 4 h in HUVECs (**Figure 1A**). Moreover, real-time PCR results showed that the mRNA levels of IL-1β, IL-8, TF, VCAM-1, and ICAM-1 were significantly elevated after aCL treatment (**Figure 1B**), implying that HUVECs were markedly injured by aCL.

Next, in order to verify whether autophagy is involved in aCL-induced cell injury, HUVECs were transfected with LC3B- RFP-GFP plasmids and then treated with aCL. The results indicated that in control cells, both red LC3-RFP and green LC3-GFP signals were mostly diffused, leading to yellow staining that is indicative of autophagosomes. In comparison, numerous LC3-RFP and LC3-GFP puncta disappeared following aCL treatment (**Figure 1C**). The results showed that aCL treatment significantly decreased the expressions of LC3 II/I and Beclin 1 and increased p62 (**Figure 1D**), indicating that autophagy was markedly suppressed by aCL treatment.

Besides, we detected the expressions of signal molecules in mTOR/S6K pathway. The results showed that the protein expressions of p-mTOR^Ser2448^ and p-S6K^Thr389^ were significantly increased following aCL treatment (**Figure 1E**), suggesting that the mTOR/S6K pathway was activated by aCL in HUVECs.

### Hyperoside Attenuated aCL-Induced Inflammatory Response in HUVECs

**Figure 2:**
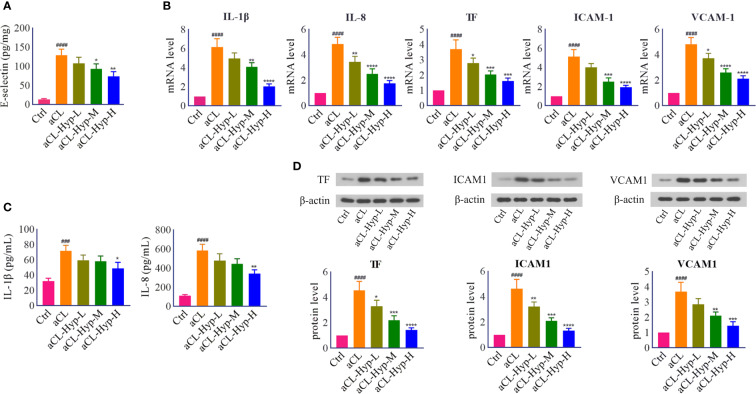
Hyperoside reduced the secretion of proinflammatory cytokines and endothelial adhesion cytokines in aCL-treated HUVECs. HUVECs were treated with 10, 20, or 50 μM of hyperoside for 24 h followed by aCL (200 μg/ml) induction for 4 h. **(A)** ELISA analysis of E-selectin. **(B)** Real-time PCR analysis of IL-1β, IL-8, TF, ICAM1, and VCAM1. **(C)** ELISA analysis of IL-1β and IL-8. **(D)** Western blot analysis of TF, ICAM1, and VCAM1 and corresponding gray values of protein bands. Data were presented as mean ± SD, n = 3. ^###^P < 0.0001, ^####^P < 0.0001 *vs.* Ctrl; ^*^P < 0.05, ^**^P < 0.01, ^***^P < 0.001, ^****^P < 0.0001 *vs.* aCL. Ctrl represented Control, Hyp represented hyperoside, L represented low dose (10 μM), M represented medium dose (20 μM), H represented high dose (40 μM).

To investigate the effect of hyperoside on aCL-induced injury, aCL-treated HUVECs were administrated with different concentrations of hyperoside. ELISA, real-time PCR, and western blot were performed to detect the expression of inflammatory response-related molecules. It turned out that hyperoside significantly decreased the expression levels of E-selectin, IL-1β, IL-8, TF, VCAM-1, and ICAM-1 in a dose-dependent manner, indicating that hyperoside effectively attenuated aCL-induced inflammatory response in HUVECs (**Figure 2**).

### Hyperoside Attenuated aCL-Induced Autophagy Inhibition in HUVECs

**Figure 3:**
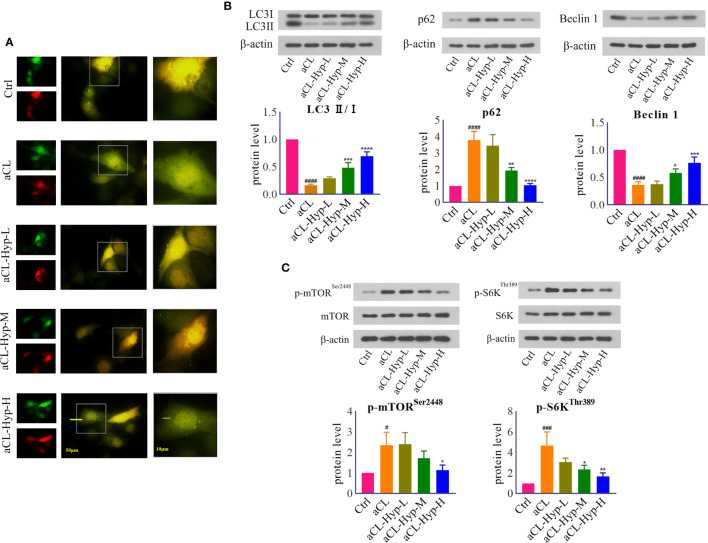
Hyperoside activated autophagy in aCL-induced HUVECs. HUVECs were transfected with LC3B-RFP-GFP plasmid, treated with 10, 20, or 50 μM of hyperoside for 24 h and then induced with aCL (200 μg/ml) for 4 h. **(A)** Fluorescence results. **(B)** Western blot analysis of LC3, p62, and Beclin 1 and corresponding gray values of protein bands. **(C)** Western blot analysis of mTOR, p-mTOR^Ser2448^, S6K, and p-S6K^Thr389^ and corresponding gray values of protein bands. Data were presented as mean ± SD, n = 3. ^#^P < 0.05, ^###^P < 0.001, ^####^P < 0.0001 *vs.* Ctrl; ^*^P < 0.05, ^**^P < 0.01, ^***^P < 0.001, ^****^P < 0.0001 *vs.* aCL. Ctrl represented Control, Hyp represented hyperoside, L represented low dose (10 μM), M represented medium dose (20 μM), H represented high dose (40 μM).

Next, we explored the effect of hyperoside on autophagy in aCL-treated HUVECs. The fluorescence results showed that hyperoside promoted the formation of autophagosomes (**Figure 3A**). The results of western blot showed that hyperoside significantly up-regulated the expressions of LC3 II/I, Beclin1 and down-regulated the expressions of p62 in aCL-treated HUVECs, indicating that hyperoside activated autophagy in aCL-treated HUVECs (**Figure 3B**). Besides, hyperoside treatment down-regulated the secretion of p-mTOR^Ser2448^ and p-S6K^Thr389^, illustrating that hyperoside inhibited the activation of mTOR/S6K signaling (**Figure 3C**).

### Hyperoside Inhibited TLR4/MyD88/NF-κB Signaling in aCL-Induced HUVECs

**Figure 4:**
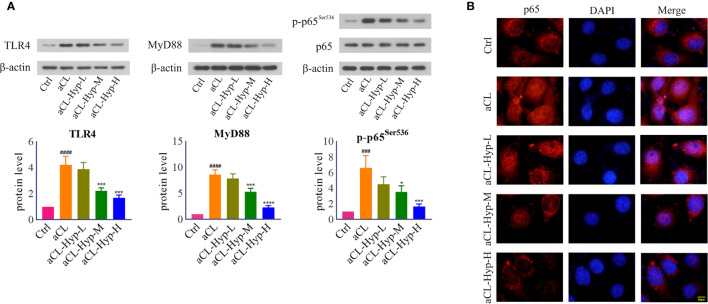
Hyperoside inhibited TLR4/MyD88/NF-κB signaling in aCL-induced HUVECs. HUVECs were treated with 10, 20, or 50 μM of hyperoside for 24 h and then induced with aCL (200 μg/ml) for 4 h. **(A)** Western blot analysis of TLR4, MyD88, p65, and p-p65^Ser536^ and corresponding gray values of protein band. **(B)** NF-κB nuclear translocation detected by immunofluorescence. Data were presented as mean ± SD, n = 3. ^###^P < 0.001, ^####^P < 0.0001 *vs.* Ctrl; ^*^P < 0.05, ^***^P < 0.001, ^****^P < 0.0001 *vs.* aCL. Ctrl represented Control, Hyp represented hyperoside, L represented low dose (10 μM), M represented medium dose (20 μM), H represented high dose (40 μM).

To uncover the potential pathways by which aCL-induced mTOR activation and autophagy suppression in HUVECs, we focused on toll-like receptor-4 (TLR4), which activates inflammatory response by inducing secretion of proinflammatory cytokines (Wu et al., 2020). Notably, treatment with aCL significantly increased the protein levels of TLR4, MyD88, and p-p65^Ser536^ (**Figure 4A**), which were dose-dependently decreased by hyperoside. Moreover, aCL obviously induced the translocation of NF-κB p65 into the nucleus in HUVECs, while hyperoside blocked the effect remarkably (**Figure 4B**).

### Hyperoside Inhibited aCL-Induced Inflammatory Response in HUVECs by Activating Autophagy

**Figure 5:**
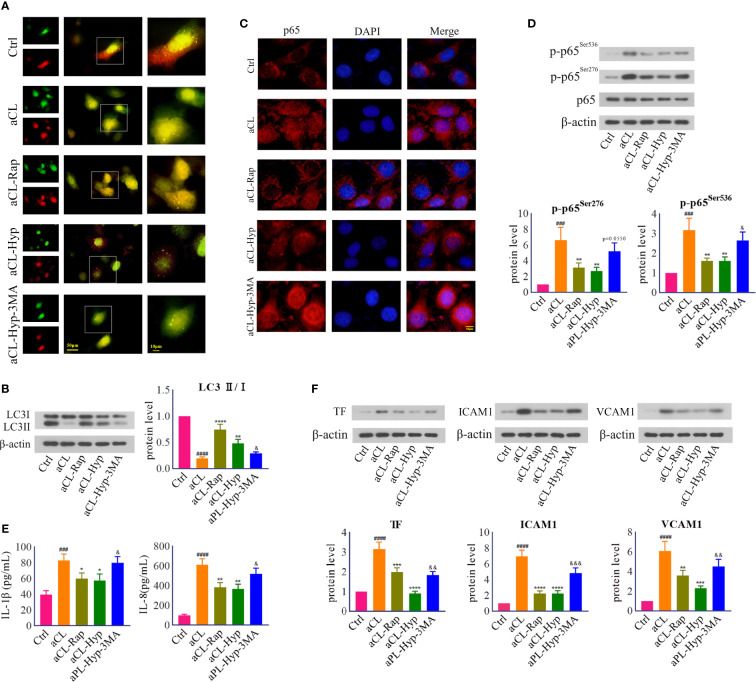
Hyperoside inhibited aCL-induced inflammatory response by activating autophagy in HUVECs. HUVECs were transfected with LC3B-RFP-GFP plasmid, treated with 10 mM of 3-MA, 50 μM of hyperoside for 24 h and then induced with aCL (200 μg/l) or 100 nM of rapamycin. **(A)** Fluorescence results. **(B)** Western blot analysis of LC3 and corresponding gray values of protein band **(C)** NF-κB nuclear translocation detected by immunofluorescence. **(D)** Western blot analysis of p65, p-p65^Ser276^, and p-p65^Ser536^ and corresponding gray values of protein band. **(E)** ELISA analysis of IL-1β and IL-8. **(F)** Western blot analysis of TF, ICAM1, and VCAM1 and corresponding gray values of protein band. Data were presented as mean ± SD, n = 3. ^###^P < 0.001, ^####^P < 0.0001 *vs.* Ctrl; ^*^P < 0.05, ^**^P < 0.01, ^***^P < 0.001, ^****^P < 0.0001 *vs.* aCL; ^&^P < 0.05, ^&&^P < 0.01, ^&&&^P < 0.001 *vs.* aCL-Hyp. Ctrl represented Control, Hyp represented hyperoside, Rap represented Rapamycin.

Furthermore, to verify whether autophagy contributes to the protection of hyperoside against aCL-induced injury, we treated aCL-induced HUVECs with rapamycin or 3-MA. It turned out that both rapamycin and hyperoside significantly attenuated aCL-induced autophagosome degradation, which was abolished by 3-MA (**Figures 5A, B**). We further detected the protein expression of p-p65 and mapped p65 translocation, and found that rapamycin and hyperoside obviously inhibited p65 nuclear translocation and its phosphorylation in aCL-treated HUVECs, while 3-MA reversed the effect (**Figures 5C, D**) partially. In addition, we detected the expressions of inflammatory cytokines IL-1β and IL-6 and endothelial adhesion molecules TF, VCAM-1, and ICAM-1 (**Figures 5E, F**). Consistently, both rapamycin and hyperoside showed therapeutic effect on aCL-induced injury of HUVECs and 3-MA reversed the inhibitory effect of hyperoside on inflammation. Collectively, the above results indicated that hyperoside attenuated aCL-induced injury of HUVECs by activating autophagy.

## Discussion

Antiphospholipid antibodies (aCL) are considered to be the cause of APS by activating endothelial cells and inducing oxidant-mediated injury (Yadalam et al., 2016). Previous studies have shown that aCL could be stimulated in an inflammatory context, leading to activation of vascular endothelial cells, monocytes, and platelets and thus thrombotic events in APS patients (Tsuchimoto et al., 2019). Thus, aCL may act as a possible cause of vascular endothelial cell injury in thrombotic events, especially in APS patients with positive aCL. However, to the best of our knowledge, little is known about the pathogenesis as well as therapeutic strategies in aCL-induced endothelial cell injury. Therefore, it is of great significance to investigate the underlying mechanisms and develop new therapeutic strategies for antiphospholipid syndrome.

Hyperoside, a major active ingredient in various Chinese traditional medicinal plants, is widely used in certain diseases due to its antioxidative and anti-inflammatory effects. Hyperoside has been proved to exert protective effects against H_2_O_2_-induced apoptosis in HUVECs (Hao et al., 2016), implying that hyperoside may be an effective compound for treating aCL-induced HUVEC injury. In this study, we confirmed that hyperoside protected HUVECs against aCL-induced injury *via* activating autophagy. We also demonstrated that the inhibition of mTOR signaling is crucial for hyperoside-mediated autophagy activation.

It has been confirmed that stimulation of monocytes and endothelial cells by aPL leads to a prothrombotic and proinflammatory state (Xia et al., 2017). To confirm the effect of aCL on cell injury, HUVECs were stimulated by aCL for different periods. E-selectin is a critical molecular marker of cell injury (Lawson, 2000). Proinflammatory cytokines IL-1β and IL-8 are two important molecules in inflammatory response (Lappas, 2017). Moreover, TF, VCAM-1, and ICAM-1 are major endothelial adhesion molecules whose levels are increased during endothelial injury (Hamilton et al., 2004). It turned out that HUVECs were markedly injured by aCL treatment, as assessed by significant decreased expressions of E-selectin, IL-1β, IL-6, TF, VCAM-1, and ICAM-1.

Autophagy, as a metabolic, cytoplasmic quality control and general homeostatic process, is primarily cytoprotective, tissue protective, and anti-inflammatory (Deretic and Levine, 2018). Many lines of evidence have demonstrated that autophagy protects against endomembrane damage triggered by various agents of endogenous or infectious origin, and prevents unnecessary or excessive inflammation (Deretic and Levine, 2018). Notably, in this study, we found that autophagy was obviously disrupted in HUVECs stimulated with aCL, as evidenced by abnormal expressions of autophagy-related molecules LC3, Beclin 1, and p62. Moreover, mTOR, an atypical serine/threonine kinase, has been confirmed to promote endogenous metabolism and suppress autophagy induction under physiological conditions (Kim and Guan, 2015). Targeting mTOR *via* regulating autophagy has become an important therapeutic strategy for inhibiting inflammation. Our data showed that aCL promoted the phosphorylation of mTOR and downstream S6K, implying the mTOR/S6K signaling pathway was abnormally activated by aCL stimulation.

To evaluate the therapeutic effect of hyperoside on aCL-induced injury, we pre-treated HUVECs with gradient concentrations of hyperoside and then with aCL for injury. Hyperoside markedly reduced the expressions of proinflammatory cytokines IL-1β and IL-6 and endothelial adhesion molecules E-selectin, TF, VCAM-1, and ICAM-1 in HUVECs in a dose-dependent manner, indicating that hyperoside exerted superior therapeutic activity in aCL-induced inflammatory response in HUVECs. In addition, hyperoside activated autophagy and suppressed mTOR/S6K pathway in aCL-induced HUVECs. Notably, we revealed the potential mechanisms underlying the association between mTOR activation and autophagy suppression. TLR4/MyD88/NF-κB pathway has been confirmed to quench intestinal inflammation and oxidative stress injury by boosting mTOR-dependent autophagy (Zhou et al., 2018). In line with the previous researches, hyperoside dose-dependently inhibited the TLR4/MyD88/NF-κB signaling pathway in aCL-induced injury of HUVECs, thereby reducing the secretion of proinflammatory and adhesion molecules.

To verify whether hyperoside exerts its anti-inflammatory effects against aCL induced injury through activating autophagy, we used 3-MA, a specific mTOR inhibitor, to block the effect of hyperoside. Meanwhile, rapamycin, a classic mTOR activator, was used as a positive control. Similar to the influence of rapamycin, hyperoside promoted autophagy activation, inhibited the phosphorylation and nuclear translocation of NF-κB p65, as well as decreased the secretion of proinflammatory and endothelial adhesion cytokines. On the contrary, 3-MA reversed the inhibitory effects of hyperoside on aCL-induced inflammation. These results demonstrated that hyperoside exerted its therapeutic effect against aCL-induced injury by specifically activating mTOR signaling-mediated autophagy.

In summary, this is the first study to link autophagy and aCL-induced injury together. This study not only newly documented mTOR-mediated autophagy as a cause of the pathogenesis of aCL-induced injury, but also offered insights into a candidate therapeutic strategy for APS treatment. It is conceivable that hyperoside may be developed as a potential therapeutic for attenuating aCL-induced vascular endothelial injury, thereby preventing the vessels from thrombosis in APS patients. Regrettably, the present study has several limitations, such as preliminary and incomprehensive data and lack of extensive preclinical experiments. Given the functional importance of autophagy in aCL-induced injury, more data and further explorations are needed to support our conclusion.

## Conclusion

Hyperoside protects HUVECs from aCL-induced injury by reducing the secretion of proinflammatory cytokines IL-1β and IL-6 and endothelial adhesion molecules E-selectin, TF, VCAM-1, and ICAM-1 *via* activating mTOR-mediated autophagy.

## Data Availability Statement

All datasets generated for this study are included in the article/supplementary material.

## Ethics Statement

The studies involving human participants were reviewed and approved by the ethics committee of First Affiliated Hospital of Henan University of Chinese Medicine. The patients/participants provided their written informed consent to participate in this study.

## Author Contributions

The study was designed by AW and YS. The manuscript was written by AW and HX. The experiments were performed by AW, HX, YS, GX, XY, JG, ZJ, and SS. The data were analyzed by AW and HX.

## Conflict of Interest

The authors declare that the research was conducted in the absence of any commercial or financial relationships that could be construed as a potential conflict of interest.
